# Defect Severity Identification for a Catenary System Based on Deep Semantic Learning

**DOI:** 10.3390/s22249922

**Published:** 2022-12-16

**Authors:** Jian Wang, Shibin Gao, Long Yu, Dongkai Zhang, Lei Kou

**Affiliations:** 1 School of Electrical Engineering, Southwest Jiaotong University, Chengdu 610031, China; 2 College of Information Engineering, Henan University of Science and Technology, Luoyang 471023, China; 3 Institute of Oceanographic Instrumentation, Qilu University of Technology (Shandong Academy of Sciences), Qingdao 266075, China

**Keywords:** catenary system, deep learning, text mining, pre-trained language model, defect severity classification

## Abstract

A variety of Chinese textual operational text data has been recorded during the operation and maintenance of the high-speed railway catenary system. Such defect text records can facilitate defect detection and defect severity analysis if mined efficiently and accurately. Therefore, in this context, this paper focuses on a specific problem in defect text mining, which is to efficiently extract defect-relevant information from catenary defect text records and automatically identify catenary defect severity. The specific task is transformed into a machine learning problem for defect text classification. First, we summarize the characteristics of catenary defect texts and construct a text dataset. Second, we use BERT to learn defect texts and generate word embedding vectors with contextual features, fed into the classification model. Third, we developed a deep text categorization network (DTCN) to distinguish the catenary defect level, considering the contextualized semantic features. Finally, the effectiveness of our proposed method (BERT-DTCN) is validated using a catenary defect textual dataset collected from 2016 to 2018 in the China Railway Administration in Chengdu, Lanzhou, and Hengshui. Moreover, BERT-DTCN outperforms several competitive methods in terms of accuracy, precision, recall, and $F_{1}$-score value.

## 1. Introduction

**Figure 1:**
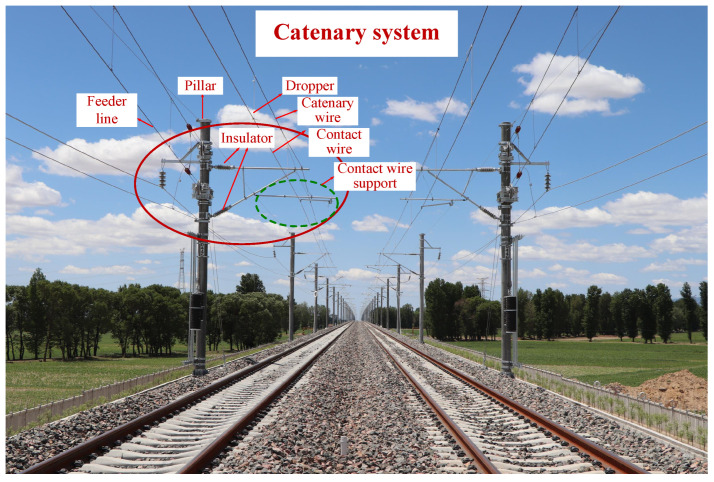
Scene of a catenary system.

A pantograph–catenary system for a high-speed railway bridges the traction power supply system and electric locomotive, whose operating conditions are of great significance for the safety and reliability performances of railway transport [1]. In particular, a catenary system is a fixed installation that consists of multiple components (as shown in Figure 1), such as the pillar, contact wire, catenary wire, feeder line, etc. Owning to the complicated failure mechanisms and dynamic outdoor environments, the operational risks of the catenary are prone to inevitably increase [2,3]. To timely mitigate the underlying risk triggers, various monitoring techniques (e.g., an automatic detection and monitoring system, also called the 6C system) and maintenance activities [4] are implemented to discover and report all potential defects of a catenary system. At the same time, a large number of catenary defect texts have been recorded and collected after manual judgment. Such textual defect text records are closely associated with the characteristics of catenary accidents and failure risks. If mined efficiently and accurately, they can provide powerful and credible information bases for discovering valuable defect knowledge and efficient defect severity identification, facilitating the subsequent defect disposal and adjustment of relevant maintenance activities [5]. Therefore, it is crucial to mine defect text records in-depth, extract critical textual semantic information, and finally identify defect severity for a catenary system.

In pace with the rapid advancement of natural language processing (NLP) technology, Chinese text mining has attracted extensive attention [6,7]. In railway systems, the associated text mining technologies have become emerging concerns and are gradually applied in several classification tasks, such as extracting traffic-relevant information, fault type classification, analyses of rail accidents, etc. Chen et al. used the long short-term memory-convolutional neural network (LSTM-CNN) with bag-of-word features to judge whether social media data are related to traffic [8]. Wang et al. applied a multichannel network with a LSTM layer and a convolution layer (MC-LSTM-Conv) and keyword fuzzy matching to detect traffic events from microblogs (i.e., traffic jams versus non-traffic jams) [9]. In [10], the support vector machine (SVM) is used to classify fault class with respect to railway signaling maintenance. Brown et al. utilized ensemble methods to predict rail accident severity [11]. To summarize, with regard to operation and maintenance defect text record data of a catenary system, text mining techniques can be applied to provide more insights into helping establish the associations between the defect event description and defect severity degree, which can ultimately improve the efficiency and accuracy of defect severity identification.

In this paper, we present an investigation to efficiently extract contextual semantic information in-depth and automatically identify catenary defect severity levels based on operation and maintenance text records. To this end, the approach integrates a combination of bidirectional encoder representation from transformers (BERT) [12] and a deep text categorization network (DTCN) with deepened Conv layers. It can abstract long-range semantic features and more global data, which are more valuable to enable defect-level classifications. Firstly, we collected catenary defect text records from 2016 to 2018 in the China Railway Administration of Chengdu, Lanzhou, and Hengshui, and analyzed the source, content, and characteristics of catenary defect texts. Afterward, Chinese word vector representations were learned based on the deep pre-trained language model BERT via the constructed catenary defect textual data set. On the basis of the generated word embedding representations, the DTCN is proposed to identify catenary defect severity (i.e., defect level category). Compared with state-of-the-art methods for text classification, the proposed BERT-DTCN can capture long-range semantic features and utilize deep semantics to distinguish the defect level. The effectiveness and superiority of our proposal are demonstrated by experimental results in terms of accuracy, precision, recall, and the $F_{1}$-score value.

The primary contributions of this paper are as follows:

- A deep semantic neural network named BERT-DTCN is proposed to effectively extract long-range semantic features and automatically identify defect severity from catenary text records.
- Different from existing domain text representation approaches that extract vector representations with bag-of-words features, we applied BERT to learn word embedding vectors and extract semantic features of domain vocabularies in defective texts. An ablation study on the constructed catenary defect text dataset validates that the generated word embedding vectors contribute to beneficial impacts on the devised text categorization model.
- Based on the obtained defect word embeddings, we used the DTCN to distinguish defect severity degree. Experimental results demonstrate that the proposed algorithm (BERT-DTCN) achieves a superior performance in the binary classification problem (level 1 defect or level 2 defect) over competitive text classification methods, which can reduce the workload of manual discrimination and improve the accuracy and efficiency of classification.

The remainder of the paper is organized as follows: Section 2 introduces the related work on text presentation and classification. Section 3 overviews the proposed catenary defect text classification approach in terms of the defect text database, word embedding presentation learning, and classification model. Section 4 presents our experimental results and analysis of the proposed model. The conclusions and suggestions for future work are drawn in Section 5.

## 2. Related Work

This study integrates methods for the defect information analysis with operational records of the catenary, word embedding representations, and catenary defect text classification. Thus, this section mainly focuses on text representation and text classification techniques.

### 2.1. Text Classification Methods

**Figure 2:**
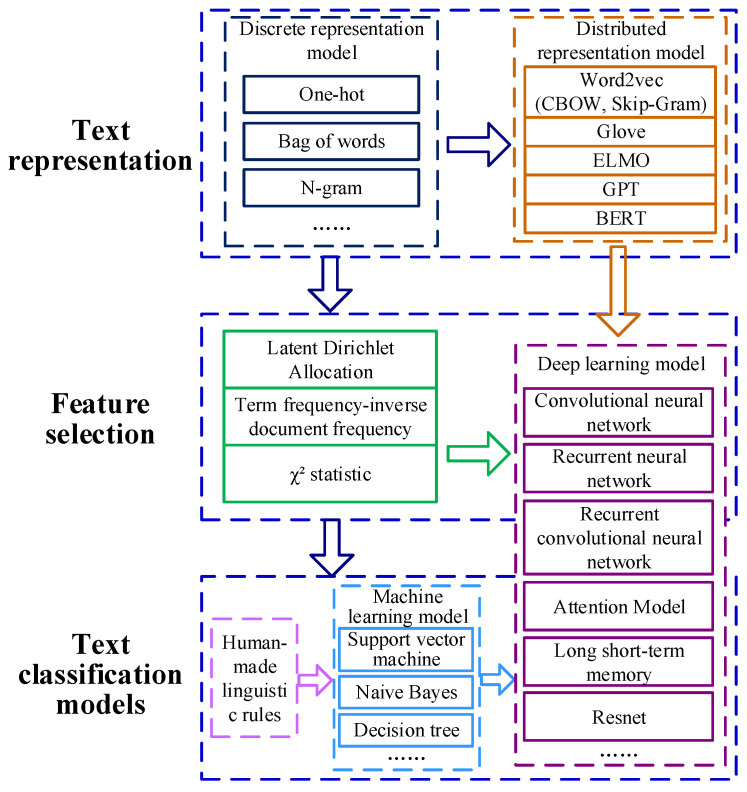
Development process of text classification.

The conventional process of text classification consists of text dataset acquisition, text preprocessing, text representation, and text classification models. Text preprocessing involves taking several measures to process textual data, including deleting stop words, word segmentation, and so on. Text representation mainly refers to the representation of converting words or sequences in a text into a form that can be recognized and handled by a machine for subsequent text classification tasks. Text classification involves utilizing the appropriate classifiers, including rule-based methods and machine learning or deep learning models, to excavate the critical features and predict the text category. In general, the development process of text classification methods can be categorized into three stages (as shown in Figure 2).

Firstly, the text classification process is investigated by human-made linguistic rules [13,14], constructing a set of rules connecting word patterns and class labels. Such human comprehensible rules can be convinced as time goes by. However, the whole constructing process consumes time and manpower and requires abundant domain knowledge [15]. However, it is not feasible to create a huge rule base for a specific domain task.

Secondly, several scholars have carried out relevant research on text mining based on traditional machine learning techniques, including naive Bayes [16], decision tree [17], SVM [18], etc., which depends on learning classification features from a pre-labeled dataset. Although machine learning-based classification methods outperform rule-based classification classifiers, they need to carefully extract classification features from text data, which play significant roles in text classifiers [19]. As a result, several feature selection approaches, such as latent Dirichlet allocation (LDA), term frequency-inverse document frequency (TF-IDF), $\chi^{2}$ statistic, mutual information, etc., are widely applied to mine topics to reduce dimensions. For instance, Wang et al. introduced a latent Dirichlet allocation (LDA) with prior knowledge to extract features [10]. Brown et al. utilized LDA to discover accident characteristics and the contributors to rail accidents [11]. However, it is obvious that these techniques do not consider the mutual position of words in text sequences and the relevant results are misleading, especially in accident texts with high similarities.

Thirdly, in pace with the emergence of deep neural networks in NLP, such deep learning techniques have been gradually utilized in text classification tasks due to their excellent performances. Various studies on text classification models, such as CNN [20], recurrent neural network (RNN) [21], recurrent convolutional neural network (RCNN) [22], attention mechanism-based bidirectional LSTM (Att-Bi-LSTM) [23], have achieved superior results toward text categorization tasks in the areas of computers, medical treatments, and electric power. Wang et al. established a CNN-LSTM-based text emotion recognition model to shed light on the developing direction of the network’s public sentiment [24]. Dai et al. used multi-sieving CNN to efficiently cope with the unbalanced microaneurysm detection problem [25]. Guerrero et al. [26] proposed a customer filtering based on the classification–regression and self-organizing map to analyze the information of inspectors’ commentaries.

### 2.2. Text Representation Models

The purpose of text presentation is to embed the words in a text into low-dimensional vectors because existing machine learning methods cannot handle text data directly. The text representation techniques (as shown in Figure 2) can be classified into discrete representation and distributed presentation. One-hot vector is one typical solution of discrete representation. It encodes an index to each word within a text corpus, and then each word can be represented by a binary vector, of which the dimension is equal to the size of this vocabulary [27]. Similar to the one-hot vector, the bag of words representation means that the vector representation of the document can be directly obtained by summing the vectors of words. Meanwhile, n-gram encodes indexes to *n* adjacent words, considering the order of words. However, such a discrete counting vector representation suffers from the dimension curse and discards the semantic relationships between words.

In the past few years, word embedding representations have been focused on; they attempt to learn low-dimensions and continuous vector representations. Word embedding represents each word with a real-value vector and uses the similarity in the vector space to represent the semantic similarity of text words, inspired by the training idea of deep learning. SkipGram and CBOW are two common word embedding methods with lower computational complexity, using a shallow neural network to perform context-based prediction [28]. Moreover, the word2vec model can compute accurate, high-dimensional word vectors from huge data sets. For instance, Li et al. [8] introduced the continuous bag-of-word (CBOW) model into generating the word embeddings and used the LSTM-CNN model to extract traffic information from 3 billion microblogs. However, such an approach neglected the distances of words (i.e., global statistical information). Hence, GloVe [29] was proposed to use the word co-occurrence and local context to learn word vectors, combining the merits of the matrix factorization and prediction-based methods. However, contextualized information is discarded in the word embedding presentation obtained by Glove, leading to great challenges of polysemy and complex syntactic features. To this end, pre-trained language models, such as embeddings from language model (ELMo) [30], generative pre-training (GPT) [31], BERT [12], etc., were investigated and explored to obtain deep contextualized word representations, integrating word embedding learning into the neural language models. In particular, with the release of BERT, it outperforms the above-mentioned models in the majority of NLP tasks, such as word embedding learning, which can efficiently capture dependencies over longer distances and excavate the actual bidirectional contextual information, improving the language understanding ability of networks via large-scale unsupervised pre-training.

Therefore, in this paper, we adopt the pre-trained language model BERT to capture long-term dependencies between text words and learning contextualized word embedding representations based on the constructed catenary defect text dataset. Moreover, motivated by the idea of ResNet [32] and the deepening of word-level convolutional neural networks (CNNs) [33], we propose a deep CNN algorithm that can efficiently capture long-range associations in text, which can extract richer semantics for domain-specific defect information identification and achieve superior performance by deepening the network without increasing computational costs by much.

## 3. Methodology

**Figure 3:**
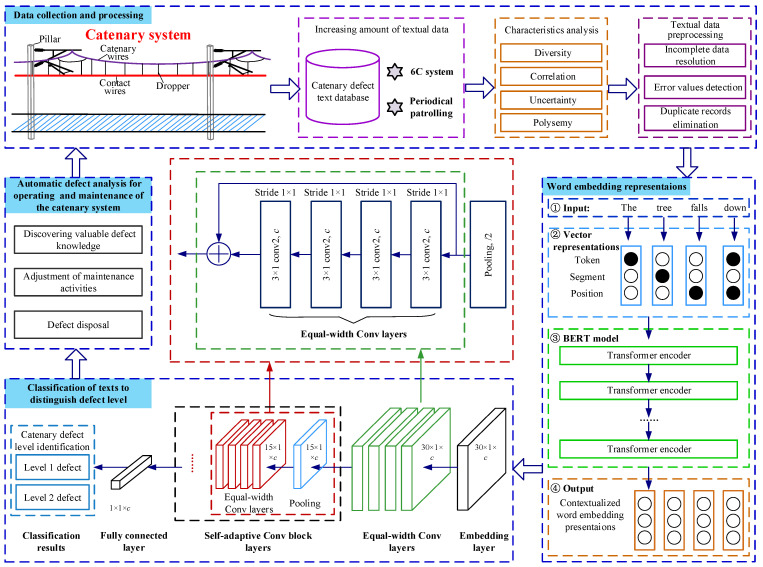
Architecture of defect severity identification for a catenary system.

As illustrated in Figure 3, the methodological framework of BERT-DTCN is mainly composed of three stages:

- **Catenary defect text database**: After obtaining the relevant catenary defect texts accumulated in the data center of the China Railway Administration during its long-time operation and maintenance, it is intended to conduct textual data prepossessing and construct the text dataset.
- **Word embedding presentations**: The BERT model projects the Chinese texts related to catenary defects into context-aware representations that can be handled and understood by machines.
- **Classification of texts to distinguish the defect level**: The DTCN module is trained to categorize the catenary defect texts by utilizing equal-width convolution and multiple convolution-residual layers with the pooling layer with stride 2 for downsampling.

### 3.1. Problem Definition

The problem tackled in this paper is defined as follows. Considering a collection of *N* defect text records that are labeled using Y=2 classes (i.e., binary problem) in a supervised manner, the catenary defect text database is denoted as $S=\left\{S_{1},S_{2},\ldots ,S_{i}\ldots ,S_{N}\right\}$ and the labels are represented by $Y=\left\{Y_{1},Y_{2}\right\}$. The supervised labeling process can be denoted as f:S→Y, where *f* is to convert the input text sequence *S* to binary vectors *Y*. The collection of all labeled training defect texts is represented as Y=f(S) [34].

The procedure of our method can be denoted as $f:S\to \hat{Y}$, which means that each defect text sequence $S_{i}\in S$ creates a label $\hat{Y_{i}}=f\left(S_{i}\right)$, $\hat{Y_{i}}\in Y$.

### 3.2. Catenary Defect Text Database

#### 3.2.1. Data Source and Text Content

Based on the 6C system and periodical patrolling, we collected the catenary defect text records from the China Railway Administration of Chengdu, Lanzhou, and Hengshui. There were nearly 45,000 defect text records available for three years (1 January 2016–31 December 2018) in total.

**Figure 4:**
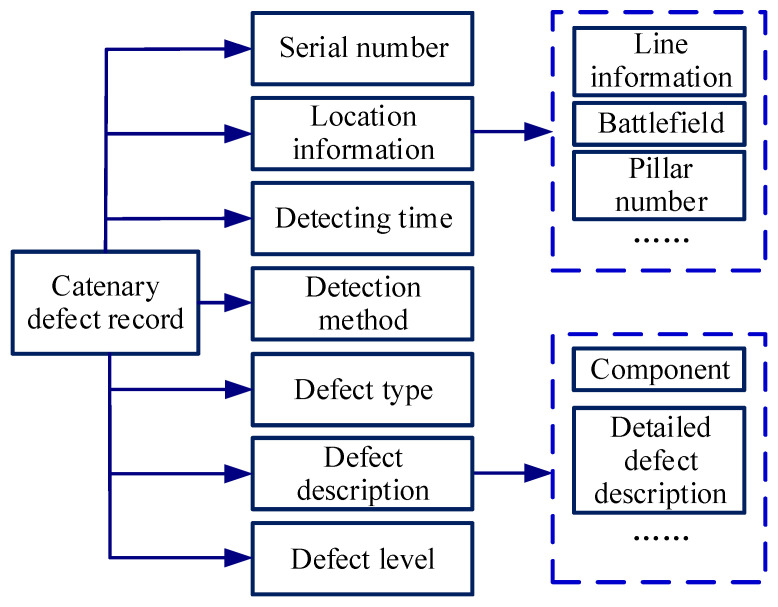
The content of the catenary defect record.

Each record can be recognized as a piece of a catenary defect, which contains the detecting time, location information, defect level, defect description, and so on, as shown in Figure 4.

#### 3.2.2. Characteristic Analysis

Compared with conventional Chinese text, catenary defect text is characterized by the following characteristics:

- Diversity. The operation and maintenance texts for the catenary system contain the time, number, unit, defect component, and defect description.
- Correlation. The operation and maintenance texts are closely linked to the railway transportation, which contains a large number of rail transit terminologies.
- Uncertainty. A great deal of defect descriptions in the catenary texts might be incomplete, noisy, fuzzy, or random.
- Polysemy. Several polysemous words in defect texts might have multiple meanings, which need to be distinguished under different semantic meanings.

#### 3.2.3. Data Processing

Given that catenary defect texts are mainly manually recorded by professional workers, the time-consuming and labor-intensive work may make recorders prone to lose their enthusiasm in long-term responses to repetitive works, leading to low-quality catenary defect text records. Hence, we conducted textual data preprocessing by the extra manual intervention. Several data cleaning methods, including incomplete data resolution, error values, duplicate records, detection, and elimination, are applied to improve the quality for the following catenary defect text classification task.

### 3.3. Word Embedding

**Figure 5:**
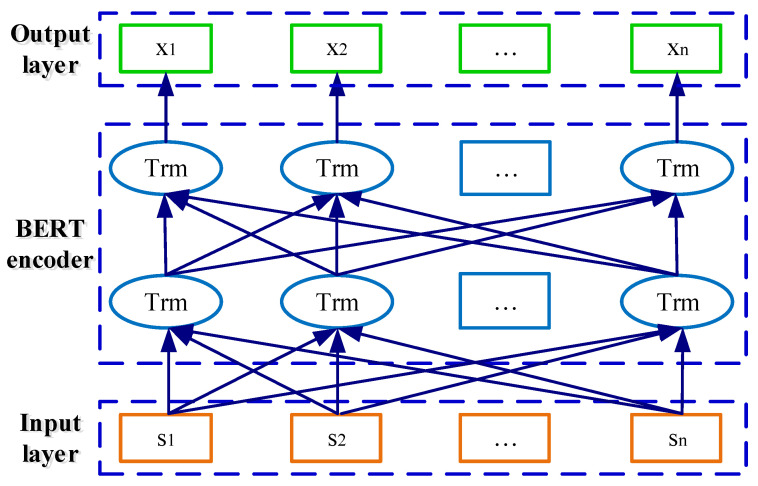
Structure of the BERT model.

To obtain the word embedding representations related to the catenary defect, the pre-trained language model BERT is adopted to learn context-aware information in this part. The structure of the BERT model is depicted in Figure 5, which consists of the input layer, BERT encoder, and output layer. It projects the Chinese input text for the catenary defect into context-aware representation [12]. Meanwhile, the generated word embedding vectors of each sequence are no more than 512 tokens. As for an input catenary defect text sequence with *n* token words $s=\left\{s_{{}_{1}},s_{{}_{2}},\ldots ,s_{{}_{n}}\right\}$, the contextualized word embedding representations in the output layer can be denoted as $x=\left\{x_{{}_{1}},x_{{}_{2}},\ldots ,x_{{}_{n}}\right\}$, with $x\in R^{nv}$.

#### 3.3.1. Input Layer

**Figure 6:**
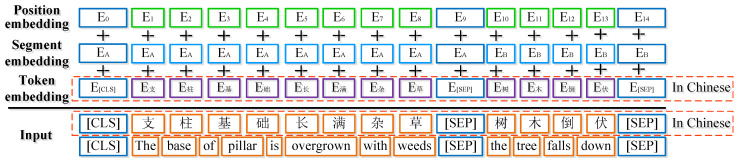
An example of the BERT input representation.

Given a token catenary defect text sequence s containing *n* words, s is represented as $s=\left\{s_{{}_{1}},s_{{}_{2}},\ldots ,s_{{}_{i}},\ldots ,s_{{}_{n}}\right\}$, where $s_{{}_{i}}\left(1\leq i\leq n\right)$ means the ith word in a Chinese text [35]. As for the input of the BERT model, special (CLS) and (SEP) tokens are added at the beginning and end of sentences respectively. In particular, the (PAD) tokens are marked at the end of the sentences to ensure their lengths are the same as the maximum sequence lengths [12]. If a single sequence consists of two sentence pairs, the sentences will be separated by the special token ((SEP)) and labeled with sentence A or sentence B, whose feature values are 0 and 1, respectively. As for each token sequence, its input representation is obtained by summing the corresponding token, segment, and position embedding. An example of the visualization of this construction is shown in Figure 6.

#### 3.3.2. BERT Encoder

**Figure 7:**
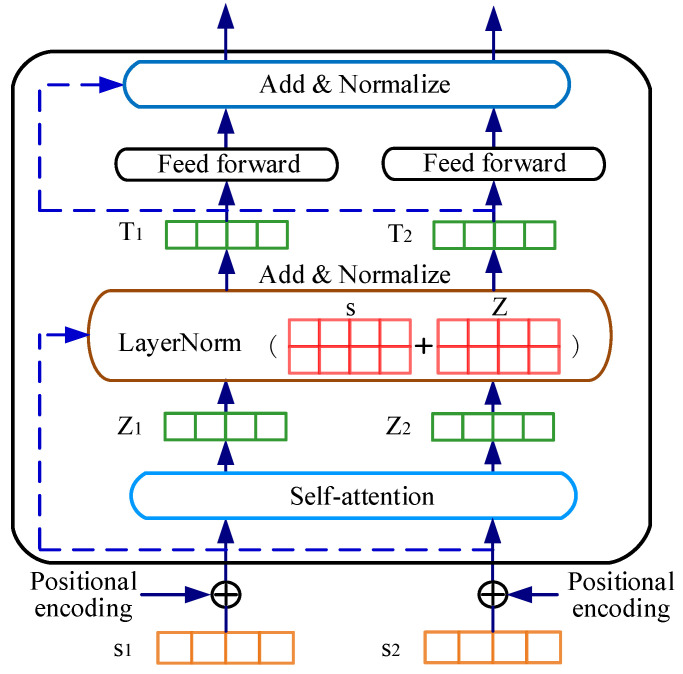
Basic structure of the transformer encoder.

The BERT used in this paper is composed of 12 transformer blocks, 768 hidden sizes, and 12 self-attention heads [36]. The basic structure of the transformer encoder is illustrated in Figure 7. The word-embedded representation of a single sequence is the token as the input of the encoder, and the positional encoding is added. The self-attention layer enables the encoder to capture the contextual information from the word when coding, which can calculate the weighted value of each word and all words. Then, the feature vector of each word is obtained, which contains the information of the whole sentence. Afterward, multiple feature vectors obtained by the multi-headed mechanism are spliced together, the dimensions of which are descended by a full connection layer. Finally, the contextualized word embedding vectors were output through the feedforward network, two residual connection layers, and a normalization layer.

**Figure 8:**
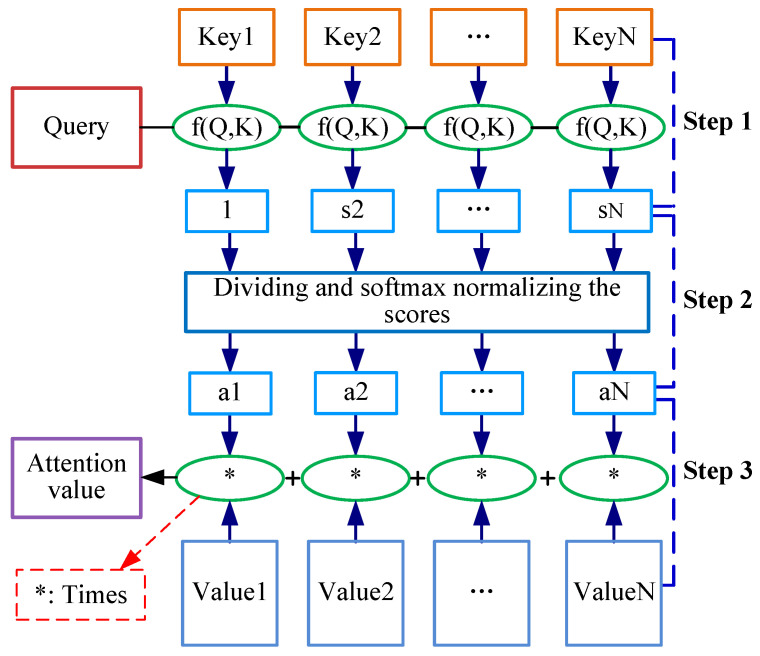
The flowchart of the self-attention layer.

The self-attention layer performs the linear transformation on the input vector to obtain a linear value, and then calculates the attention weight, as depicted in Figure 8. The calculation of self-attention consists of three steps:

(1) Creating three vectors (i.e., a query vector, a key vector, and a value vector) from each of the encoder’s input vectors and obtaining a weighted score by calculating the dot products of the query with all Keys. It can be calculated as:
  (1) $$f\left(Q,K_{i}\right)=QK_{i}^{T}$$
(2) Dividing the scores by scaling factor $\sqrt{d_{k}}$ and then normalizing the scores through a softmax operation. It can be represented as:
  (2) $$a_{i}=\mathrm{softmax}\left(\frac{f(Q,K_{i})}{\sqrt{d_{k}}}\right)=\frac{\exp(\frac{f(Q,K_{i})}{\sqrt{d_{k}}})}{\sum_{j}\exp\left(\frac{f(Q,K_{i})}{\sqrt{d_{k}}}\right)}$$
(3) Multiplying each value vector by the softmax scores and summing up the weighted value vectors. It can be defined as:
  (3) $$\mathrm{Attention}\left(Q,K,V\right)=\sum_{i}a_{i}V_{i}$$

where *Q, K, V* refer to the query, key, and value matrix, and $\sqrt{d_{k}}$ represents the scaling factor.

Thus, such word embedding presentations not only contain the meanings of the words themselves, but also capture the relationships among words. Compared with the traditional word embedding methods, BERT-based embedding can obtain the representation of text with rich semantic information.

### 3.4. Deep Text Categorization Network

The DTCN involves taking the obtained word embedding representations as the input, categorizing the catenary defect texts, and distinguishing the severity levels using defect text records. Motivated by the idea of ResNet and deep pyramid CNN [33], we propose a deep learning-based defect text categorization network called DTCN that adopts the structures of deep CNNs, which can achieve the superior performance by deepening the network without increasing computational costs by much. As discussed previously, its structure is depicted in Figure 3, which consists of four components, namely, the embedding layer, equal-width convolution layers, stacking of convolution blocks (equal-width convolution layers and a shortcut) interleaved with max-pooling layers with stride 2 for downsampling, and a fully connected layer.

#### 3.4.1. Embedding Layer

We used a convolution layer to transform the learned word embedding into the feature maps, the dimensions of which are the *number of filters* × (seq_length-2). It is essentially a feature extractor that encodes semantic features in a given dimension (related to the number of filters and lengths of text sequences), in which words with similar semantics also have closer Euclidean or cosine distances. The convolution operation is to move a filter over the text sequence matrix (input map) and compute the dot products. In DTCN, to learn more sophisticated features, two filters are used to convolve the input word embedding vectors, and all feature vectors are concatenated into a three-dimensional convolution feature map.

Let $x_{{}_{i}}\in R^{nv}$ represent the *v*-dimensional word vector with respect to the ith word in a sentence with *n* words. The input map of DTCN can be denoted as

(4) $$x_{{}_{1:n}}=x_{{}_{1}}⊕x_{{}_{2}}⊕\cdots ⊕x_{{}_{n}}$$

where ⊕ refers to concatenation operator. $x_{{}_{i:i+j}}$ represents the concatenation of words $x_{{}_{i:i+j}}=x_{{}_{i}},x_{{}_{i+1}},\cdots ,x_{{}_{i+j}}$. The filter $w\in R^{mv}$ function involves computing a new feature in the window of *m* words. Thus, a feature $c_{i}$ is produced from a window of words $x_{{}_{i:i+m-1}}$ by

(5) $$c_{i}=f\left(w\cdot x_{{}_{i:i+m-1}}+b\right)$$

where b∈R is a bias and *f* is an activation function.

The feature map is generated from each possible window of words in the sentence $\left\{x_{{}_{1:m}},x_{{}_{2:m+1}},\cdots ,x_{{}_{n-m+1:n}}\right\}$. It is represented as

(6) $$c=\left[c_{1},c_{2},\cdots ,c_{n-m+1}\right]$$

where $c\in R^{n-m+1}$.

#### 3.4.2. Downsampling with the Number of Feature Maps Fixed

The increasing number of feature maps cannot improve the accuracy, but only increase the computation time substantially;l thus, the DTCN adopts equal-width convolution to enrich the semantic representation by keeping the same number of channels. After equal-width convolution layers, the convolution block (equal-width convolution layers and max-pooling with size 3 and stride 2) (as shown in Figure 3) is performed with the number of channels (also called filters) that are fixed. As a result, the length of the text sequence vectors is halved, and then the computation time of each convolutional layer is reduced by half. Moreover, the number of convolution blocks is automatically determined by the length of the defect text sequence. Therefore, the total computation time is bounded by the computation time of a convolution block. In addition, downsampling with stride 2 can efficiently double the coverage of the convolution kernel, which is computationally efficient in representing long-range associations and more global information.

#### 3.4.3. Shortcut Connections with Pre-Activation

Due to the saturated accuracy and rapid degradation with the network depth increasing, there are great difficulties in training the deeper neural networks, such as higher training errors with more layers, and vanishing/exploding gradients [32]. To better train the deep networks, the DTCN model uses additive shortcut connections with identity mappings in [37]: z+f(z), where *f* represents skipped convolution layers with pre-activation. In particular, pre-activation means that activation is done before weighing. Thus, the convolution layers of the DTCN can be computed by Wσ(x)+b, where x refers to a small region (overlapping with each other) of word embedding vectors at each location, σ(·) is a component-wise nonlinear activation, and weights W and biases b are needed to be trained. In DTCN, activation σ(·) is set to σ(x)=max(x,0). In addition, linear weighting Wσ(x)+b with pre-activation eases the training of deep networks [38,39].

In our training process, the training set includes two types of labels, i.e., “0” (severity level 1) and “1” (severity level 2). The BERT-DTCN is an overall framework that is trained automatically. The details of the training algorithm are shown in Algorithm 1. The BERT-DTCN is trained by minimizing the cross-entropy loss function based on the defect text dataset. The loss function of the classifier for the BERT-DTCN is denoted as follows:

(7) $$Loss_{c}=-\sum_{1}^{n}\left[y_{i}\log\hat{y_{i}}+\left(1-y_{i}\right)\log\left(1-\hat{y_{i}}\right)\right]$$

where $y_{i}$ means the actual label of the input defect text sequence *i*; $\hat{y_{i}}$ is the probability vector corresponding to the output of the BERT-DTCN of the input defect text sequence *i*; and *n* is the number of training samples,
**Algorithm 1** Pseudocode for training the BERT-DTCN.**Require:** 
$X_{s}$: training set for the BERT-DTCN, including the constructed defect text dataset and labels; $N_{c}$: number of classifier-training iterations per mini-batch.1:**for** the number of training iterations **do**2:    Sample mini-batch of *m* examples from the training set $X_{s}$;3:    **for** $i=1\to N_{c}$ **do**4:        Update the BERT-DTCN by minimizing the loss: $Loss_{c}$5:    **end for**6:**end for**

## 4. Experiment Results and Analysis

To facilitate the performance evaluation of the BERT-DTCN, we investigated some training protocols and comparisons with competing text classification approaches on the catenary defect text dataset in this section.

We obtained nearly 45,000 catenary defect text records from 2016 to 2018 in the China Railway Administration of Chengdu, Lanzhou, and Hengshui. After data cleaning and filtering, we labeled 11,106 catenary defect text records that were applied in the catenary defect severity identification, and the proportions of the training, verification, and test defect text dataset were set to 0.7, 0.15, and 0.15, respectively. Table 1 lists the details of the catenary defect text dataset that we used for the experiments.

We applied the BERT model to generate word embedding presentations for the subsequent defect information extraction and defect level discrimination. In our experiments, we found that a majority of defect text records were concise and brief, and then the maximum length of word sequences was set to 32 (i.e., the padding size). The zeros after each sequence were padded until the length reached 32. Thus, the number of convolution blocks could be determined and was set to 4. Afterward, the general Chinese Bert language model “bert-base-chinese” was used to learn word embeddings, and then the generated word vector was a 768-dimensional vector, which was equal to the number of hidden units. The obtained word embedding vectors of each sequence can be represented by a 32×768 matrix, which could be fed into our classification model.

In addition, all programs were implemented under the PyTorch framework. The main configuration of the computer was a 1080Ti graphics card, Intel Xeon E5 v3, with 32G of memory [3].

### 4.1. Training Protocol

In this section, some training protocols of the BERT-DTCN are investigated. We focus on two key parameters: the number of convolution layers in equal-width convolution layers (called $N_{cl}$) and the number of output channels (filters) (called $N_{f}$) in the convolution. In particular, because the equal-width convolution layers were applied in BERT-DTCN, the number of input channels was equal to that of the output channels.

$N_{cl}$ and $N_{f}$: The $N_{cl}$ determines the depth of neural networks, which allows each lexeme to contain more and longer contextual information. The $N_{f}$ is closely associated with the number of feature maps, which restricts the dimensions of the semantic space and determines the size of the output probability map. We trained the BERT-DTCN with the hyperparameter settings listed in Table 2 under various $N_{cl}$ and $N_{f}$, and the results are given in Table 3.

We found that $N_{cl}$ and $N_{f}$ contributed to some differences in the classification performance and training time. It demonstrates that a network with a deeper structure and large sizes of feature maps is not necessary to achieve better performance. The computation burden increases with the increasing number of $N_{cl}$ and $N_{f}$; however, the whole computation time is indistinguishable. This is because after the convolution block, max-pooling with the number of feature maps fixed is performed, and then the computation time for each convolution layer is halved. Thus, the total computation time is almost the same. According to the results, we set the $N_{cl}=4$ and the $N_{f}=140$. Therefore, the detailed structure parameters of the BERT-DTCN are set as listed in Table 4.

### 4.2. Ablation Study

There are two critical modules that work cooperatively in the BERT-DTCN model, namely, BERT-based word embedding presentation and deepening of CNN-based text classification (DTCN). In order to validate the effectiveness of the BERT, we compare the performance of BERT-DTCN and DTCN in this section. The relevant parameter settings of the DTCN and BERT-DTCN are the same as listed in Table 2 and Table 4. We trained two networks with the same training protocols based on the constructed dataset.

The most intuitive evaluation index of the classification problem is used, i.e., accuracy rate, the percentage of correctly classified samples in the total number of samples. Except for the accuracy rate, three evaluation indexes related to text categorization were adopted, namely, precision (*P*), recall (*R*), and the $F_{1}$-score ($F_{1}$) [12,35]. In this part, we take the accuracy rate as the primary evaluation of the text classification model, and the $F_{1}$, *P*, and *R* as the auxiliary indicators.

**Figure 9:**
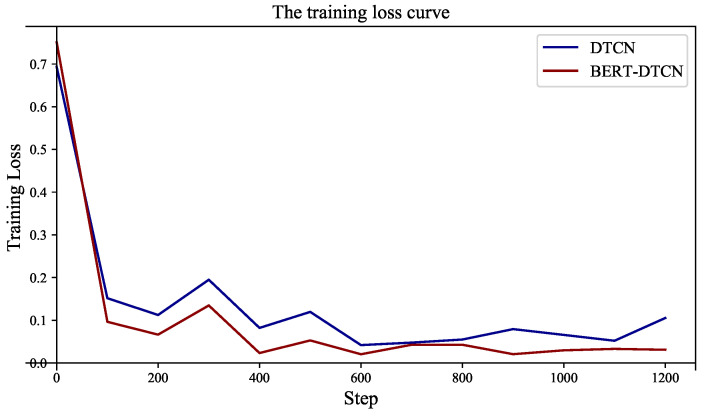
The training loss of DTCN and BERT-DTCN.

**Figure 10:**
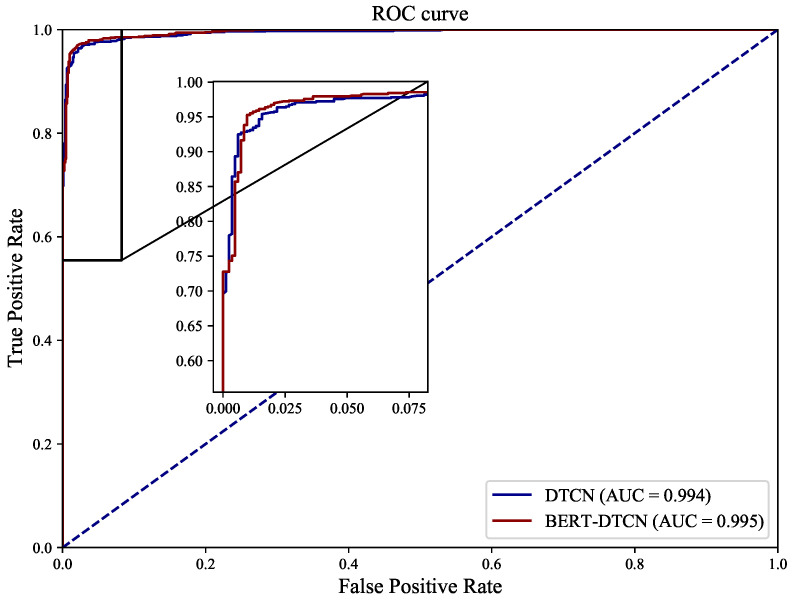
The ROC curves for DTCN and BERT-DTCN.

**Figure 11:**
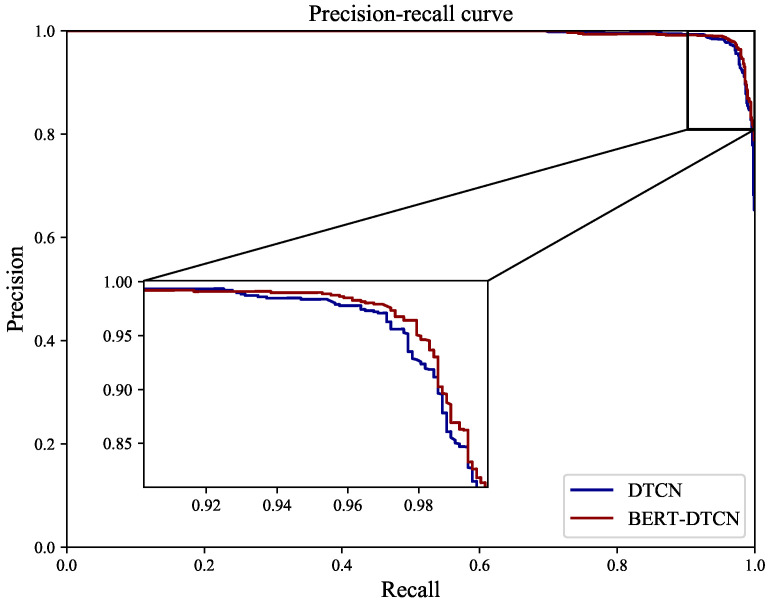
The PR curves for DTCN and BERT-DTCN.

As illustrated in Table 5, regardless of which category the defect text is in, the BERT-DTCN achieves superior results in all of the elevation metrics (Acc, *P*, *R*, and $F_{1}$). Moreover, the overall accuracy of the catenary text classification reaches up to 97.40%. Compared with the DTCN, the macro *P*, *R*, $F_{1}$, and accuracy of BERT-DTCN are all improved by 0.42%. At each level of the catenary defect text dataset, the BERT-DTCN model achieves improvements ranging from 0.36% and 0.53%. In addition, the training loss curves in Figure 9 demonstrate that the BERT-DTCN converges faster and achieves better performances with fewer steps over DTCN. The ROC curve displays the trade-off between the true positive rate or sensitivity (proportion of positive tuples that are recognized) and the false-positive rate (proportion of negative tuples that are incorrectly recognized as positive) for DTCN and BERT-DTCN. The ROC curve in Figure 10 shows that the BERT-DTCN has a larger area under the ROC curve than that of DTCN, with a better severity level classification performance in the catenary defect text. Moreover, the PR curve in Figure 11 shows that the BERT-DTCN has both high precision, and high recall, characterizing the superior effectiveness of classification performance.

To summarize, it is obvious that the BERT-based word embeddings have significant positive impacts on the classification performance of the DTCN. This is because the word embedding vectors obtained by BERT can accurately represent the semantic features of catenary defect vocabulary and capture contextual information, improving the performance of the catenary defect severity identification to a certain extent. Hence, we conclude that the BERT can learn contextual semantic information, which reduces the interference brought by the non-standard parts in the defect texts and contributes to beneficial effects on the text categorization performance.

### 4.3. Classification Performance Comparison

To better evaluate the classification performance of BERT-DTCN, we compare our method with state-of-the-art deep learning models for text classification based on the constructed catenary defect text database.

The following baselines are adopted to validate the effectiveness and efficiency of the BERT-DTCN model:

**CNN**: A method for sentence-level classification tasks based on CNN [20].

**RNN**: RNN for multitask learning [21].

**RCNN**: A method of extracting contextual information for text classification based on RCNN [22].

**FastText**: A fast training model with a large corpus [40].

**Att-Bi-LSTM**: A word-level text categorization model based on Att-Bi-LSTM [23].

**Transformer**: A model architecture that enables global dependencies captured based on an attention mechanism [36].

We trained these networks with the same dataset and applied accuracy, *P*, *R*, and $F_{1}$ to evaluate the performances of these competing approaches.

As shown in Table 6, these comparative results illustrate that the BERT-DTCN achieves better performances in all elevation metrics. On the constructed catenary defect text database, the macro *P*, *R*, $F_{1}$, and accuracy of BERT-DTCN reach up to 97.40%. This is because the BERT-DTCN can capture richer features through specific downsampling and learned word embedding, enhancing the representation of defect texts. Our model called BERT-DTCN outperforms competing models that are widely used in the text classification task. Moreover, the CNN, RCNN, and Att-Bi-LSTM also achieve superior performance in the catenary defect text classification in terms of macros *P*, *R*, $F_{1}$, and accuracy based on the constructed defect text dataset.

**Figure 12:**
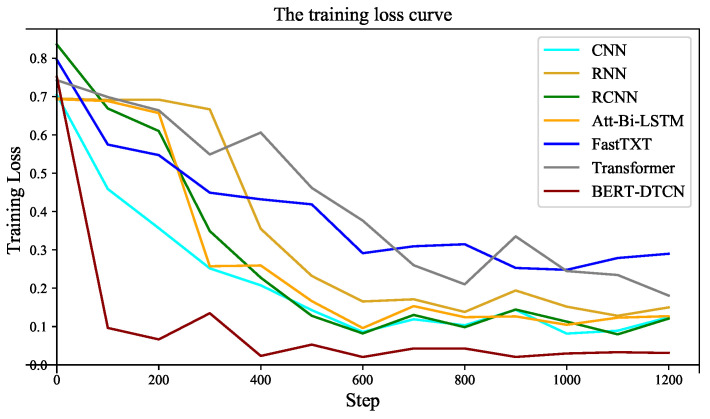
The training loss of BERT-DTCN and competing models.

**Figure 13:**
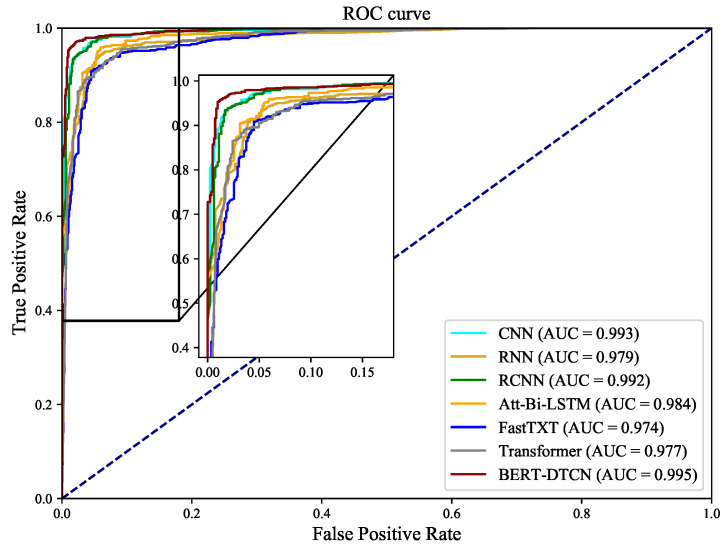
The ROC curves for BERT-DTCN and competing models.

**Figure 14:**
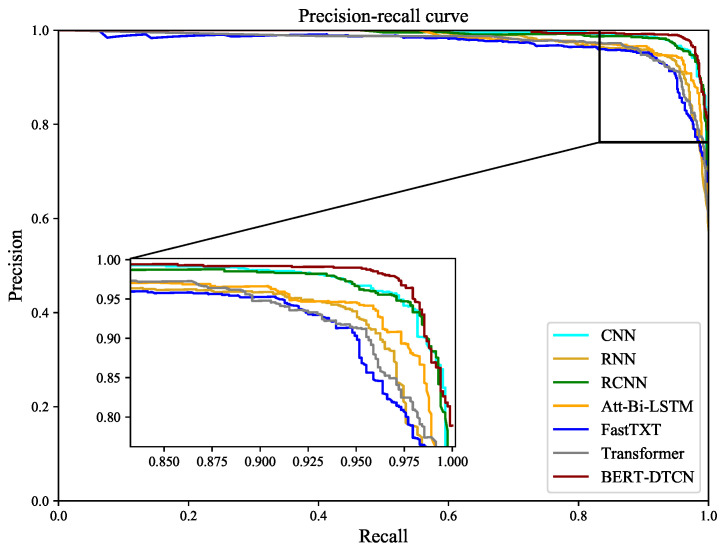
The PR curves for BERT-DTCN and competing models.

Compared with the competing methods, the training loss curves in Figure 12 demonstrate that the BERT-DTCN converges faster and achieves better performances with fewer steps. As depicted in Figure 13 and Figure 14, the ROC and PR curves show that the BERT-DTCN can enhance performance over several state-of-the-art models. In general, the BERT-DTCN model achieves superior performance in the catenary defect level classification, which validates the effectiveness of the DTCN and BERT-DTCN. It is obvious that the DTCN and BERT-DTCN with the strategy of deepening the network can capture the global information in the text, achieving a fairly superior performance.

In addition, the BERT-DTCN used word embedding presentations pertaining to BERT to initialize word embeddings in the deep text categorization network and then feed it as training proceeded (distinguishing the defect severity level). The DTCN module in BERT-DTCN can be regarded as a deep extension of shallow CNN, sharing region embedding enhancement with diverse unsupervised embeddings. Based on the experimental results of the DTCN and CNN in Table 5 and Table 6, the DTCN with deepened networks can perform improvements in capturing more global information over CNN, which has proven the conclusions in [33], i.e., the added depth is indeed useful.

## 5. Conclusions

In this paper, we reported on the emerging text mining based on catenary defect records collected in the operation and maintenance of the catenary. We investigated the deep semantic learning method to automatically identify the severity level of the catenary defect. Firstly, we analyzed and summarized the characteristics of the catenary text, including diversity, correlation, uncertainty, and polysemy, and established the text dataset for the deep semantic learning-based defect text classification model. Different from counting vector representations obtained by discrete representative approaches, we applied the pre-trained language model BERT to learn contextual word embedding vectors. At the training phase, BERT-DTCN was trained by the cross-entropy loss to extract relevant defect information. Then, the classifier can learn how to distinguish between the severity level 1 defect and severity level 2 defect, i.e., the complex defect information extraction problem was transformed into a simple classification problem. We thoroughly analyzed the impacts of the training protocol and word embedding presentations obtained by BERT and compared the BERT-DTCN with other competing methods. The ablation experiments showed that the word embedding vectors obtained by BERT contributed to positive effects on the superior performance of the developed DTCN, which demonstrated the effectiveness of our classification model BERT-DTCN on the constructed catenary defect text dataset. Comparative experiments showed that BERT-DTCN outperformed the competing deep learning methods, which can effectively represent long-range associations in the catenary defect texts and extract global semantic information with deepened networks. Moreover, we found that the strategy of deepening the network can improve the classification performance to a certain extent. The number of filters and the depth of the network were two significant parameters for the deep networks. The proposed model can be applied in the operation and maintenance of a catenary system to extract defect information and categorize defect severity.

As for catenary defect record-based text mining, various issues and challenges need to be further explored, which can contribute to fruitful and beneficial results for the safe operation and maintenance of a catenary system. The extended catenary maintenance corpora and terminology dictionaries might enhance the performance to a certain extent. In addition, imbalanced data are other unsolved problems in this domain-specific task, which have adverse effects on the performances of existing categorization algorithms. In the future, these NLP tasks, such as imbalanced learning for catenary defect texts, BERT-based named entity recognition in Chinese catenary defect texts, and knowledge graph construction for the health management of a catenary system [6], deserve to be investigated in depth.

## Figures and Tables

**Table 1:** The details of the catenary defect text classification dataset.

Dataset Name	Classes of Defect Level	Training Set	Verification Set	Test Set
Catenary Defect Text	2	7611	1652	1653

**Table 2:** The Hyperparameter settings of BERT-DTCN.

Hyperparameter	Setting	Hyperparameter	Setting
Learning rate	0.00005	Padding size	32
Optimizer	Adam	Embedding	768
Batch size	128	Epoch	20

**Table 3:** Accuracy and training time under different $N_{cl}$ and $N_{f}$.

$N_{\mathrm{cl}}$	$N_{f}$	Acc/%	Training Time
Two equal-width convolution layers	120	96.73	17 min 25 s
	130	96.49	17 min 15 s
	140	96.49	17 min 12 s
	150	96.67	17 min 19 s
	160	97.10	17 min 19 s
Three equal-width convolution layers	120	96.61	17 min 13 s
	130	97.04	17 min 46 s
	140	97.34	17 min 26 s
	150	97.22	17 min 54 s
	160	97.22	18 min 31 s
Four equal-width convolution layers	120	96.67	18 min 01 s
	130	97.22	17 min 29 s
	140	**97.40**	17 min 29 s
	150	97.16	17 min 16 s
	160	96.43	17 min 18 s

^bold^ is with best classification performance.

**Table 4:** The detailed structure parameters of the BERT-DTCN.

Layer		Input Size	Kernel Size	Stride	Output Size	Number
Input	BERT	—	—	—	32×768×1	1
Embedding layer	Conv2	32×768×1	3×768	1	30×1×140	1
Equal-width convolution layers	Padding1_1	30×1×140	—	—	32×1×140	1
	Conv2_1	32×1×140	3×1	1	30×1×140	
	Padding1_2	30×1×140	—	—	32×1×140	
	Conv2_2	32×1×140	3×1	1	30×1×140	
	Padding1_3	30×1×140	—	—	32×1×140	
	Conv2_3	32×1×140	3×1	1	30×1×140	
	Padding1_4	30×1×140	—	—	32×1×140	
	Conv2_4	32×1×140	3×1	1	30×1×140	
Convolution block	Padding2	30×1×140	—	—	31×1×140	4
	Max-pooling	31×1×140	3×1	2	15×1×140	
	Padding1_1	15×1×140	—	—	17×1×140	
	Conv2_1	17×1×140	3×1	1	15×1×140	
	Padding1_2	15×1×140	—	—	17×1×140	
	Conv2_2	17×1×140	3×1	1	15×1×140	
	Padding1_3	15×1×140	—	—	17×1×140	
	Conv2_3	17×1×140	3×1	1	15×1×140	
	Padding1_4	15×1×140	—	—	17×1×140	
	Conv2_4	17×1×140	3×1	1	15×1×140	
Output	Fully connected layer	140	—	—	2	1

**Table 5:** Comparative results between BERT-DTCN with DTCN.

Model	Severity Level 1 (827)			Severity Level 2 (826)			Macro Average			Acc/%
	*P*/%	*R*/%	$F_{1}$/%	*P*/%	*R*/%	$F_{1}$/%	*P*/%	*R*/%	$F_{1}$/%	
DTCN	96.75	97.22	96.98	97.20	96.73	96.97	96.98	96.98	96.98	96.98
BERT-DTCN	**97.23**	**97.58**	**97.40**	**97.57**	**97.22**	**97.39**	**97.40**	**97.40**	**97.40**	**97.40**

^bold^ is with best classification performance.

**Table 6:** Comparison of BERT-DTCN with competing approaches on the constructed catenary defect text database.

Model	Severity Level 1 (827)			Severity Level 2 (826)			Macro Average			Acc/%
	*P*/%	*R*/%	$F_{1}$/%	*P*/%	*R*/%	$F_{1}$/%	*P*/%	*R*/%	$F_{1}$/%	
CNN	96.01	96.01	96.01	96.00	96.00	96.00	96.01	96.01	96.01	96.01
RNN	94.94	92.29	93.95	93.12	95.04	94.07	94.03	94.01	94.01	94.01
RCNN	96.70	95.53	96.11	95.57	96.73	96.15	96.13	96.13	96.13	96.13
Atti-Bi-LSTM	95.81	94.07	94.94	94.17	95.88	95.02	94.99	94.98	94.98	94.98
FastText	93.41	92.50	92.95	92.57	93.46	93.01	92.99	92.98	92.98	92.98
Transformer	94.04	91.66	92.84	91.85	94.19	93.01	92.95	92.92	92.92	92.92
BERT-DTCN	**97.23**	**97.58**	**97.40**	**97.57**	**97.22**	**97.39**	**97.40**	**97.40**	**97.40**	**97.40**

^bold^ is with best classification performance.

## Data Availability

Not applicable.
